# IMPATIENT-qPCR: monitoring SELEX success during in vitro aptamer evolution

**DOI:** 10.1007/s00253-024-13085-7

**Published:** 2024-04-04

**Authors:** Ann-Kathrin Kissmann, Grigory Bolotnikov, Runliu Li, Franziska Müller, Hu Xing, Markus Krämer, Kay-E. Gottschalk, Jakob Andersson, Tanja Weil, Frank Rosenau

**Affiliations:** 1 Institute of Pharmaceutical Biotechnology, Ulm University, Albert-Einstein-Allee 11, 89081 Ulm, Germany; 2 Max Planck Institute for Polymer Research Mainz, Ackermannweg 10, 55128 Mainz, Germany; 3 Institute of Experimental Physics, Ulm University, Albert-Einstein-Allee 11, 89081 Ulm, Germany; 4 AIT Austrian Institute of Technology GmbH, Giefinggasse 4, 1210 Vienna, Austria

**Keywords:** Aptamer, SELEX, qPCR

## Abstract

**Abstract:**

SELEX (Systematic Evolution of Ligands by Exponential enrichment) processes aim on the evolution of high-affinity aptamers as binding entities in diagnostics and biosensing. Aptamers can represent game-changers as constituents of diagnostic assays for the management of instantly occurring infectious diseases or other health threats. Without in-process quality control measures SELEX suffers from low overall success rates. We present a quantitative PCR method for fast and easy quantification of aptamers bound to their targets. Simultaneous determination of melting temperatures (*T*_m_) of each SELEX round delivers information on the evolutionary success via the correlation of increasing GC content and *T*_m_ alone with a round-wise increase of aptamer affinity to the respective target. Based on nine successful and published previous SELEX processes, in which the evolution/selection of aptamer affinity/specificity was demonstrated, we here show the functionality of the IMPATIENT-qPCR for polyclonal aptamer libraries and resulting individual aptamers. Based on the ease of this new evolution quality control, we hope to introduce it as a valuable tool to accelerate SELEX processes in general.

IMPATIENT-qPCR SELEX success monitoring. Selection and evolution of high-affinity aptamers using SELEX technology with direct aptamer evolution monitoring using melting curve shifting analyses to higher *T*_m_ by quantitative PCR with fluorescence dye SYBR Green I.

**Key points:**

*• Fast and easy analysis.*

*• Universal applicability shown for a series of real successful projects.*

**Graphical Abstract:**

**Figure:**
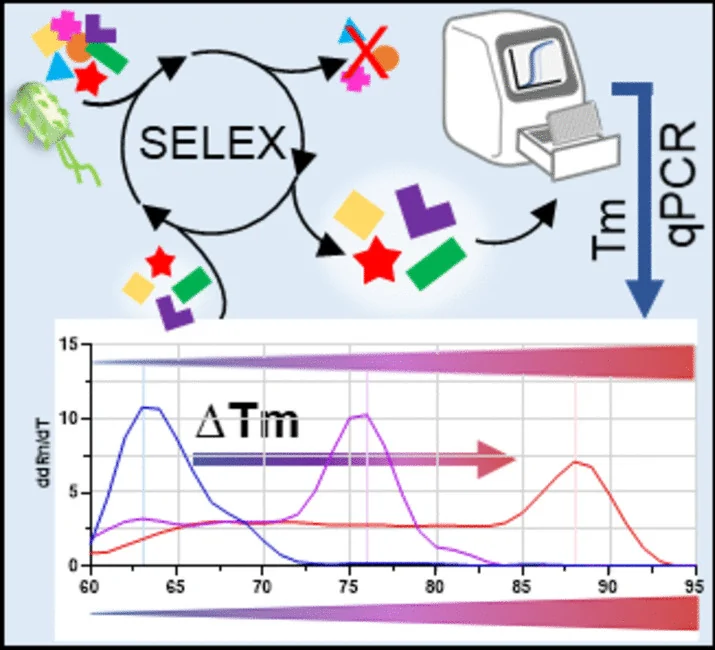

## Introduction

Aptamers are short, single-stranded oligonucleotides, which have become an increasingly attractive alternative to antibodies or their derivatives since their introduction in 1990 (Ellington and Szostak 1990; Tuerk and Gold 1990). Aptamers form complex three-dimensional structures and thereby show high specificity and affinity for their dedicated targets ranging from small molecules and chemical compounds such as metal ions (Qu et al. 2016), proteins (Bayat et al. 2018; Kissmann et al. 2022a), whole cells, and microorganisms (Sefah et al. 2010; Kubiczek et al. 2020; Raber et al. 2021; Xing et al. 2021, 2022a, 2022b; Kneißle et al. 2022; Zhang et al. 2023) to more complex targets such as cancerous tissues (Zhong et al. 2019; Li et al. 2021) or even plant parts (Kissmann et al. 2022b). However, unlike antibodies, aptamers are physically and chemically very stable, can be regenerated without losing their function, are easy to modify, can be synthesized in large quantities at relatively low cost in vitro, and have a low overall immunogenicity (Chopra et al. 2014; Ku et al. 2015; Sharma et al. 2017). Their molecular evolution is done completely in vitro from synthetic random oligonucleotide libraries with a typical sequence space of 10^12^–10^16^ distinct sequences entering the iterative selection and enrichment process called SELEX (Systematic Evolution of Ligands by EXponential enrichment) (Ellington and Szostak 1990; Tuerk and Gold 1990).

This process can be designed to meet specific requirements of intended applications, allowing high-affinity aptamers to be specifically selected against an enormous variety of targets. This selection technology consists of repeating sequential steps of target binding and removal of unbound oligonucleotides, followed by elution, amplification, and purification of the selected aptamers (Stoltenburg et al. 2005; Sefah et al. 2010; Zhuo et al. 2017). During this process, the sequence diversity of the aptamer pool decreases until only molecules with the highest binding affinity remain, which typically are then finally analyzed and characterized with respect to their specific binding properties and performance (Ellington and Szostak 1992). The SELEX process normally takes weeks to months and is, while running, always accompanied by uncertainty and not every selection is successful (McKeague and DeRosa 2012; Ouellet et al. 2015). Standard SELEX procedures only have a success rate of about 30%, whereas adapted variants with careful monitoring of the aptamer evolution on a round to round basis with the use of modified libraries have a chance of success of about 75% (McKeague and DeRosa 2012; Ochsner et al. 2014). The multistep, cyclical SELEX process can theoretically be continued until only a single sequence remains. However, as many high-affinity aptamer candidates as possible should be isolated and without insight into the course of the process, it is difficult to determine the right time to stop the selection; hence, it is essential to monitor the progress of selection using fast and simple, but at the same time precise and reliable techniques to significantly increase the chances of a finally successful and fast selection (McKeague and DeRosa 2012; Pearson et al. 2022). In turn the complexity of the scientific task to perform a fast, reliable, and finally successful efficient SELEX may be discouraging and the reason for the undeservedly slow development of the field in the first 25 years of aptamer research with a remarkable acceleration in the last few years in parallel with the invention of monitoring techniques for the SELEX success.

## Material and methods

### Quantitative PCR

For analysis of the aptamer libraries, quantitative PCR (qPCR) was performed with the eluates of the respective SELEX rounds using the qTOWER^3^G touch (Analytik Jena, Jena, Germany) and the fluorescent dye SYBR Green I (Sigma-Aldrich, St. Louis, MO, USA) under the following thermal cycling conditions: an initial step of 3 min at 94 °C, followed by 40 cycles of 30 s at 94 °C, 30 s at 56 °C, and 10 s at 72 °C. A master mix was prepared for each reaction batch. Therefore, a 5 × Herculase II Reactions buffer (Agilent Technologies, Inc., Santa Clara, CA, USA), a Herculase II Fusion DNA Polymerase (Agilent Technologies, Inc., Santa Clara, CA, USA), and a 100-mM dNTP mix (Agilent Technologies, Inc., Santa Clara, CA, USA) were used. Also, 100-mM unmodified primers (forward primer: 5′-TAG GGA AGA GAA GGA CAT ATG AT-3′; reverse primer: 5′-TCA AGT GGT CAT GTA CTA GTC AA-3′) (biomers.net GmbH, Ulm, Germany) were used. For each assay, 0.003761 pmol of the respective aptamer DNA was employed. All amplification reactions were performed in triplicates, including no-template controls (NTC) in each run to check for impurities.

### Absolute quantification

For absolute quantification, a standard straight line of a synthetic aptamer library of known concentration was established using a decadic dilution series in the range of 0.0001–1 ng. For this, the threshold cycle (*C*_t_) value was plotted against the relative concentrations. Based on the slope, the efficiency could be calculated using the formula: *Efficiency* (%) = (10 (1/slope) − 1) × 100. Subsequently, the *C*_t_ value could be determined using qPCRsoft 4.0 software, and thus, the amount of DNA immediately after the SELEX rounds in the samples could be calculated. The increase in DNA amount with increasing number of SELEX rounds could be described with an exponential growth (Nonlinear regression fit, Exponential growth equation).

### Melting curve analysis

At the end of the amplification protocol, a melting curve analysis was performed consisting of 40 melting cycles starting at 60 °C with increases of 1 °C per cycle to a temperature of 95 °C to differentiate whether the reaction resulted in the formation of a specific PCR product or whether non-specific by-products were generated. Linear regression analysis was performed to test the relationship between ddRn/dT (change in fluorescence divided by change in temperature) and the different SELEX rounds.

### Structure prediction

The secondary structure of the selected aptamers was predicted using the DNA folding program mfold. This involves modeling the likely secondary structures, taking into account folding temperature and buffer conditions. This prediction is based on the highest calculated thermodynamic stability of the structures.

## Results

Since the sample volume including the eluted aptamers after each SELEX round is very limited and represents a unique ensemble of distinct sequences, post-SELEX characterization methods must be extremely sensitive, if reamplification is intended to be avoided ensuring the actual diversity of the sequence space. Fluorescence-based techniques have been introduced allowing sensitive affinity measurements; however, methods like the FluMag- or FluCell-SELEX require the original target samples, which in the case of complex recombinant proteins or even patient-derived cancer tissues represent truly precious specimen (Stoltenburg et al. 2005; Kubiczek et al. 2020; Raber et al. 2021). Thus, alternative techniques are urgently required to immediately measure the quantity of aptamers eluted from these targets in each SELEX round and to estimate the successful evolution of aptamers with potentially higher affinity towards the dedicated target structure. The principle function of the aptamer 3D structure is the coordination of molecular binding sites in the 3D space, which include the phosphate negative charge providing electrostatic interactions of the nucleotide backbone and, probably more important, sites for hydrogen bonding. In nucleic acids these are the nucleobases which offer two or three hydrogen bonds, respectively (two for adenine and thymine or three for cytosine and guanine). It is not a presumptuous thought to expect aptamers evolving towards higher affinity, i.e., formation of stronger bonding to the target structure, to contain relatively higher numbers of GC rather than AT sequences. In consequence successful evolution should be accompanied by an increase of the melting temperature for the desired improved aptamers in the respective library, which thus can serve as an indirect but easy measure to monitor the directed evolution of aptamer affinity. Quantitative PCR (qPCR) as a workhorse method in molecular biology to quantitatively and sensitively measure relative concentrations of nucleic acids not only allows the mentioned determination of actual aptamer concentrations after each SELEX round, but also classically allows to precisely measure the melting temperature of the resulting PCR products (Wilhelm and Pingoud 2003; Hong et al. 2014).

**Fig. 1:**
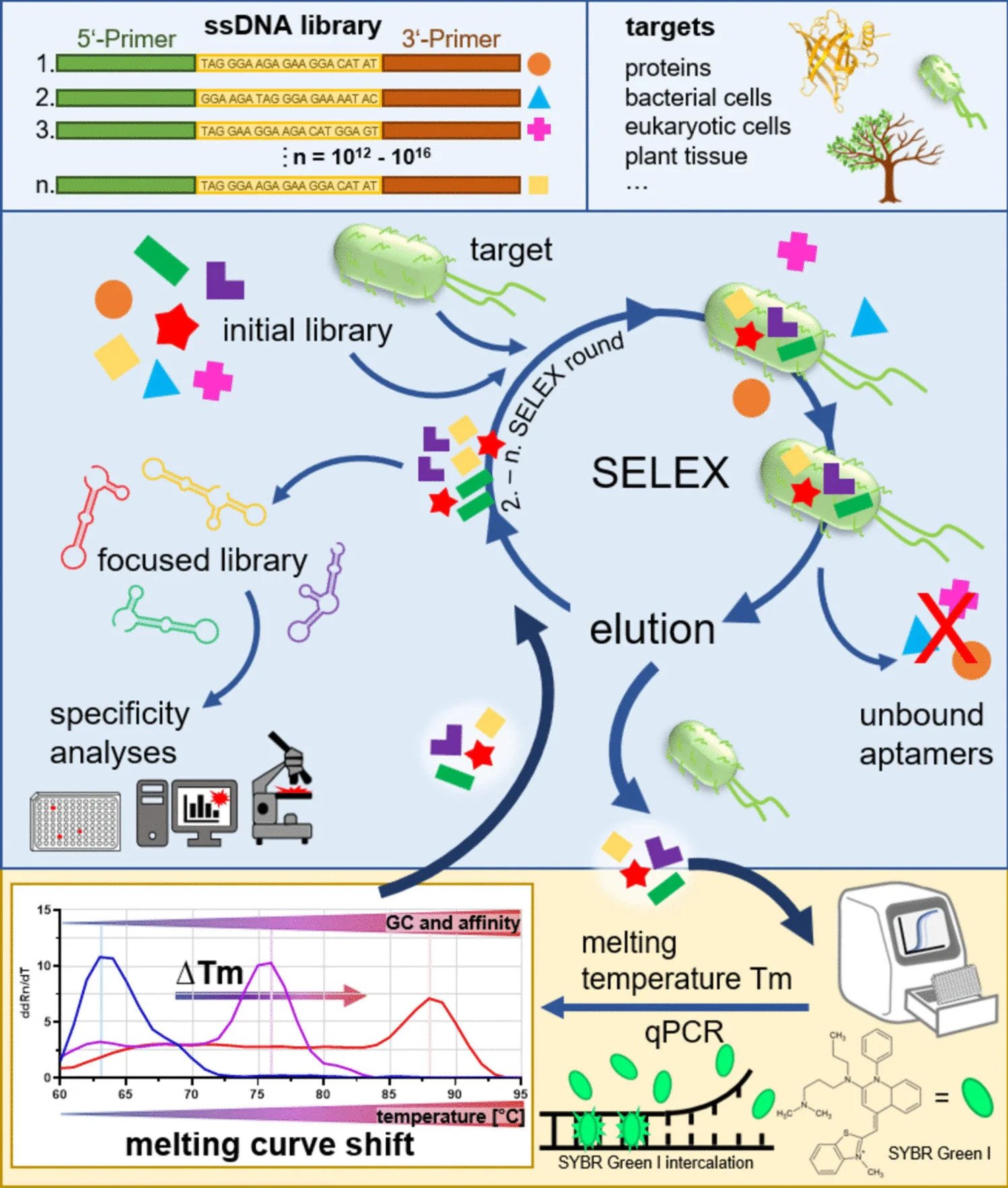
Selection of high-affinity aptamers using SELEX and their characterization using modern bioanalytical techniques. A randomized single-stranded aptamer library (approximately 10^12^–10^16^ distinct sequences) is incubated with a dedicated target molecule, binding and non-binding aptamers are separated by simple washing, and after elution and amplification of the binder aptamers focused (i.e., specific) libraries were obtained. Direct aptamer evolution monitoring uses melting curve shifting analyses to higher *T*_m_ by quantitative PCR with fluorescence dye SYBR Green I. By repetition of this procedure with increasing selection pressure a specific polyclonal aptamer library is evolved, which can carefully be monitored and afterward characterized by, e.g., suspension assays or fluorescence microscopy

We have successfully used qPCR-based quantification of aptamers eluted over the course of SELEX processes in our most recent previous projects on the development of polyclonal libraries against major yeast pathogens like *Candida auris*, *C. albicans*, and *C. parapsilosis* as well as against plant roots of *Arabidopsis thaliana* and *Hordeum vulgare* and the human gut bacterium *Rikenella microfusus* (Kneißle et al. 2022; Kissmann et al. 2022b; Zhang et al. 2023). Melting studies for assessment of library diversity in SELEX have been described by several research groups in the aptamer community (Schütze et al. 2010; Avci-Adali et al. 2013; Vanbrabant et al. 2014; Mencin et al. 2014; Luo et al. 2017; Kong et al. 2017). However, appreciating the ease of this qPCR analysis for monitoring sequence evolution and SELEX success, we decided to share our experience in more detail with the community to encourage interested researchers to valiantly and unconcernedly start SELEX projects with complex and/or precious biological target structures. Although the principle behind the method is not new the reliability of the techniques depends on the amount of comprehensive and comparative studies. In contrast to the mentioned existing previous studies on the use of qPCR in SELEX based on only single individual targets we here experimentally compare a range of examples from previous and successful SELEX studies. These additional projects involved different targets including retinol binding protein 4 (RBP4), the opportunistic pathogenic bacterium *Pseudomonas aeruginosa*, and probiotic strains from the human gut including *Akkermanisa muciniphila*, *Blautia producta*, *Roseburia intestinalis*, and *Parabacteroides distasonis*, which have delivered either functional polyclonal aptamer libraries and/or individual bioinformatically selected aptamers. We here strengthen the principal applicability of qPCR for monitoring SELEX success (Fig. 1). Moreover, we dare to suggest the denotation IMPATIENT-qPCR for this PCR variant allowing the “melt**I**ng-te**MP**er**AT**ure-sh**I**ft based **E**volution mo**N**i**T**oring” (IMPATIENT) of aptamers as a fast and simple quality control of SELEX enabling a data-based estimation of promising end-points of aptamer evolution towards high-affinity molecular target recognition.

**Fig. 2:**
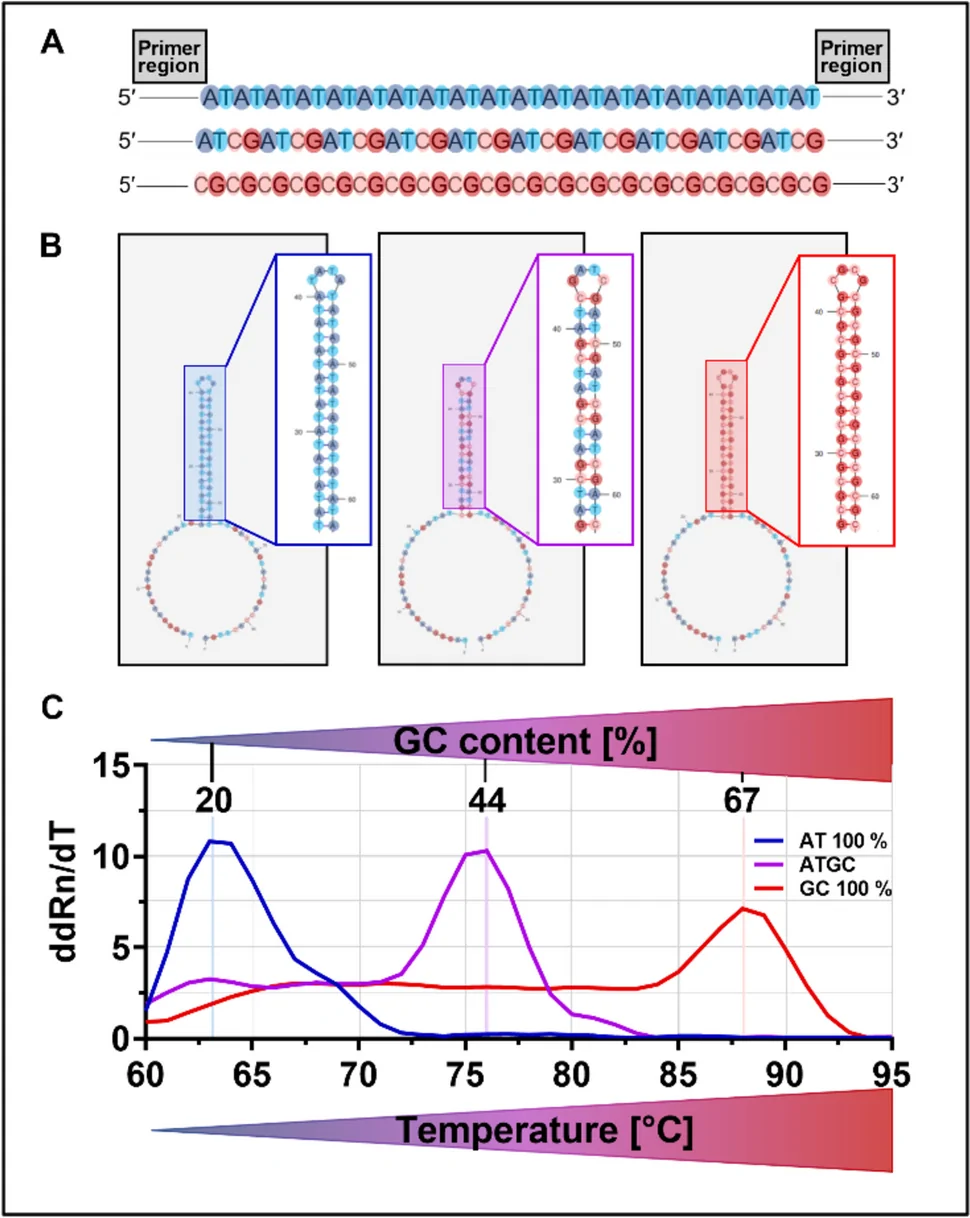
qPCR-based evolution analysis of AT (100%), GC (100%), and ATGC (25% of each nucleotide) aptamers with known GC content. **A** AT, GC, and ATGC aptamer sequence without primer regions. The individual nucleotides were colored. **B** Predicted secondary structure of AT (− 11.28 kcal/mol), GC (− 35.88 kcal/mol), and ATGC (− 21.03 kcal/mol) aptamers using mfold software. The stem loop of the aptamers were enlarged and the individual nucleotides were colored. **C** Melting temperature in dependence on GC content. Melting curves show an increase in melting temperature for aptamers with higher GC content. ddRn/dT describes the change in fluorescence divided by the change in temperature. All experiments were performed as triplicates (*N* = 3) using qTOWER.^3^G Touch (Analytik Jena GmbH, Jena, Germany)

Increasing affinity during SELEX is in principle accompanied by a gain of GC content in the random part of the aptamer sequences. Thus, the idea that determining the melting temperature of the respective eluted (focused) aptamer library after each selection round may deliver information about the status of the process is in principle self-evident. We have demonstrated previously this coherency for the SELEX towards functional libraries against *Candida* spec. and plant roots (Kneißle et al. 2022; Kissmann et al. 2022b) and wanted to prove this effect for the other SELEX projects of our laboratory. As a calibration we referenced the classical melting curve determination via incorporation (and release) of SYBR Green into double-strand DNA fragments during qPCR with model aptamers consisting of concatemeric sequence blocks of pure AT, pure GC, or ATGC in the random sequence area excepting only the primer binding sites from this scheme (Fig. 2A). Structure prediction revealed models for each sequence forming stable stem loops of the respective sequence blocks only excluding the primer binding sites (Fig. 2B). It was thus not surprising that melting temperatures for these sequences were highest for the GC-rich sequence and intermediate for the AT oligonucleotide (Fig. 2C).

**Fig. 3:**
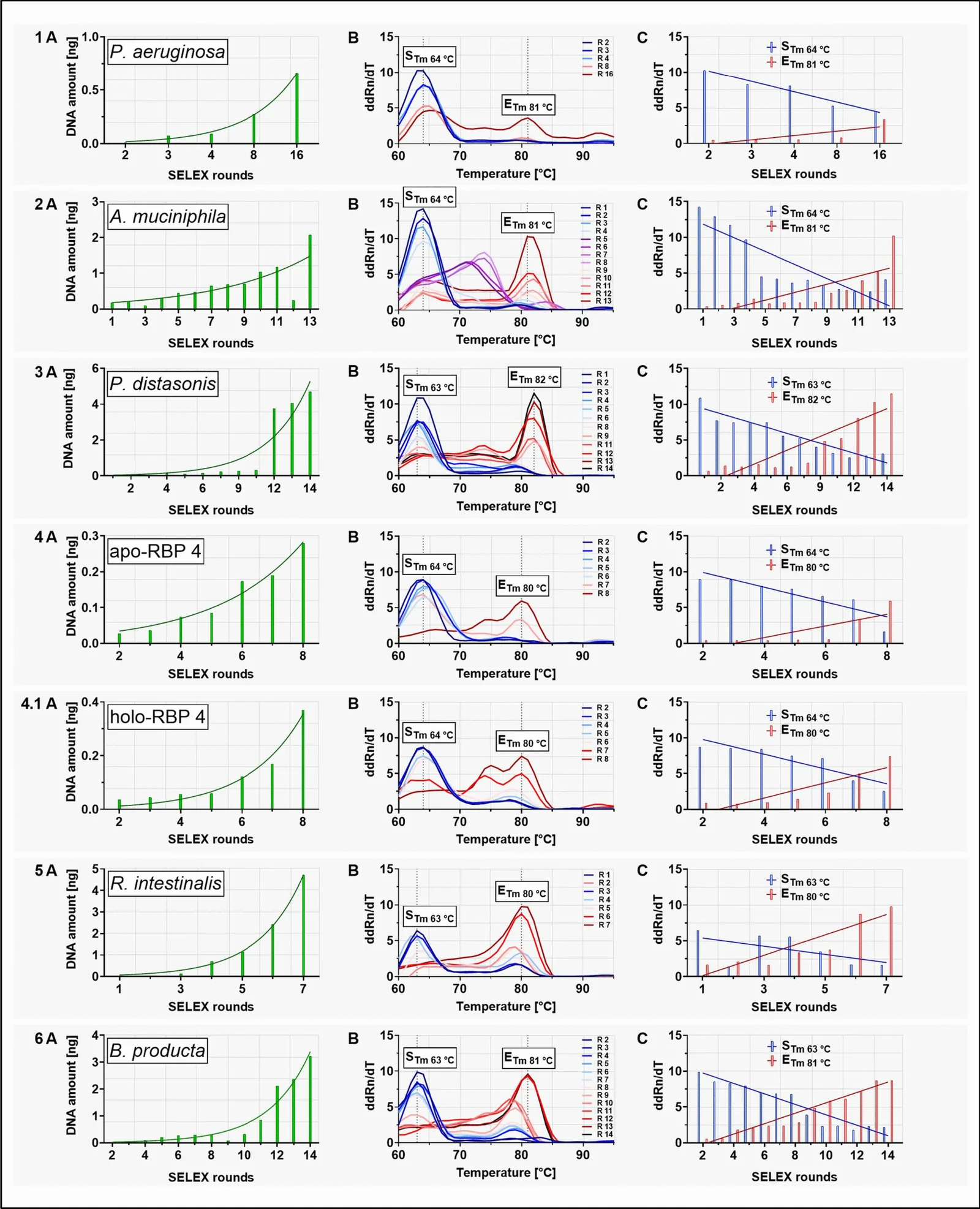
Quantitative PCR-based evolutionary analysis of aptamer libraries from different already published projects. (1) *P. aeruginosa* aptamer library from round 2 to 16, (2) *A. muciniphila* aptamer library from round 1 to 13, (3) *P. distasonis* aptamer library from round 1 to 14, (4) apo-retinol binding protein 4 aptamer library from round 2 to 8, (4.1) holo-retinol binding protein 4 aptamer library from round 2 to 8, (5) *R. intestinalis* aptamer library from round 1 to 7, and (6) *B. producta* aptamer library from round 2 to 14. For each aptamer library (1–6) the DNA concentrations in the eluates immediately after the SELEX rounds (**A**); melting curves with two main temperature peaks, *S*_Tm_ at the beginning of the SELEX process and *E*_Tm_ at the end of the process (**B**); and peak shift analyses for *S*_Tm_ and *E*_Tm_ (**C**) are shown. ddRn/dT describes the change in fluorescence divided by the change in temperature. The increase in DNA amount with increasing number of SELEX rounds could be described with an exponential growth (Nonlinear regression fit, Exponential growth equation (A)). Linear regression analysis was performed to test the relationship between ddRn/dT (change in fluorescence divided by change in temperature) and the different SELEX rounds (Linear regression fit (C)). All experiments were performed in triplicate (*N* = 3) using qTOWER.^3^G Touch (Analytik Jena GmbH, Jena, Germany) and the fluorescent dye SYBR Green I (Sigma-Aldrich, St. Louis, MO, USA)

The already published polyclonal aptamer libraries serving as examples of successful SELEX processes in this study have been shown to possess binding affinities and specificities conform with the respective defined application requirements for specific binding of their targets and include the whole Cell-SELEX processes against *P. aeruginosa* and *A. muciniphila* SELEX from which also individual aptamers have been isolated and characterized (Kubiczek et al. 2020; Raber et al. 2021), which were included for testing the IMPATIENT-qPCR here. The samples analyzed here were original libraries of the designated SELEX rounds eluted directly after each selection. IMAPTIENT-qPCR revealed two major effects of successful SELEX, which can serve as quality control measures during the molecular evolution of aptamers directed towards better binding. One of the key indicators is the quantity of aptamer DNA eluted from the SELEX target in each round, which was in fact observed for all processes and indicates an increase of the affinity in the libraries (Fig. 3 1A–6A). The melting curves of the PCR DNA double-strand fragments originating from the highly diverse mixture of aptamers within each round were analyzed along a temperature scale from 60 to 95 °C and unveiled a shift of the average temperature maxima (i.e., the average melting temperatures (*T*_m_) of the respective library) running to higher *T*_m_ through the course of the SELEX rounds with maxima of the melting temperature peaks for the start and end libraries at 63–64 °C (*S*_Tm_) and 80–82 °C (*E*_Tm_) (Fig. 3 1B–6B). Since the library templates of IMPATIENT-qPCR samples typically resemble mixtures of different sequences descending from billions at the start to several (hundred) thousands of individual sequences at the end of the SELEX the melting curves tend to have a broad distribution of melting temperatures with, in our experiments, these two major peaks representing the average of individual melting temperatures present in the actual library. Consequently, this allows quantification of the relative ddRn/dT values at the peak temperatures, which then deliver a measure of the *T*_m_ shift for each SELEX round with ddRn/dT decreasing at *S*_Tm_ and simultaneously increasing at *E*_Tm_ (Fig. 3 1C–6C). Moreover, the melting temperatures of the individual aptamers isolated from SELEX against *P. aeruginosa* and *A. muciniphila* predominantly match *E*_Tm_ of the polyclonal libraries (Table 1). The aptamer C4R2 against *P. aeruginosa* deviated with a *T*_m_ of only 76 °C and thus represents an interesting example for the applicability of the IMPATIENT qPCR, since it was shown to be significantly less sensitive and affine in the original study compared to the other individual aptamers and the polyclonal library (Kubiczek et al. 2020).

**Table 1:**
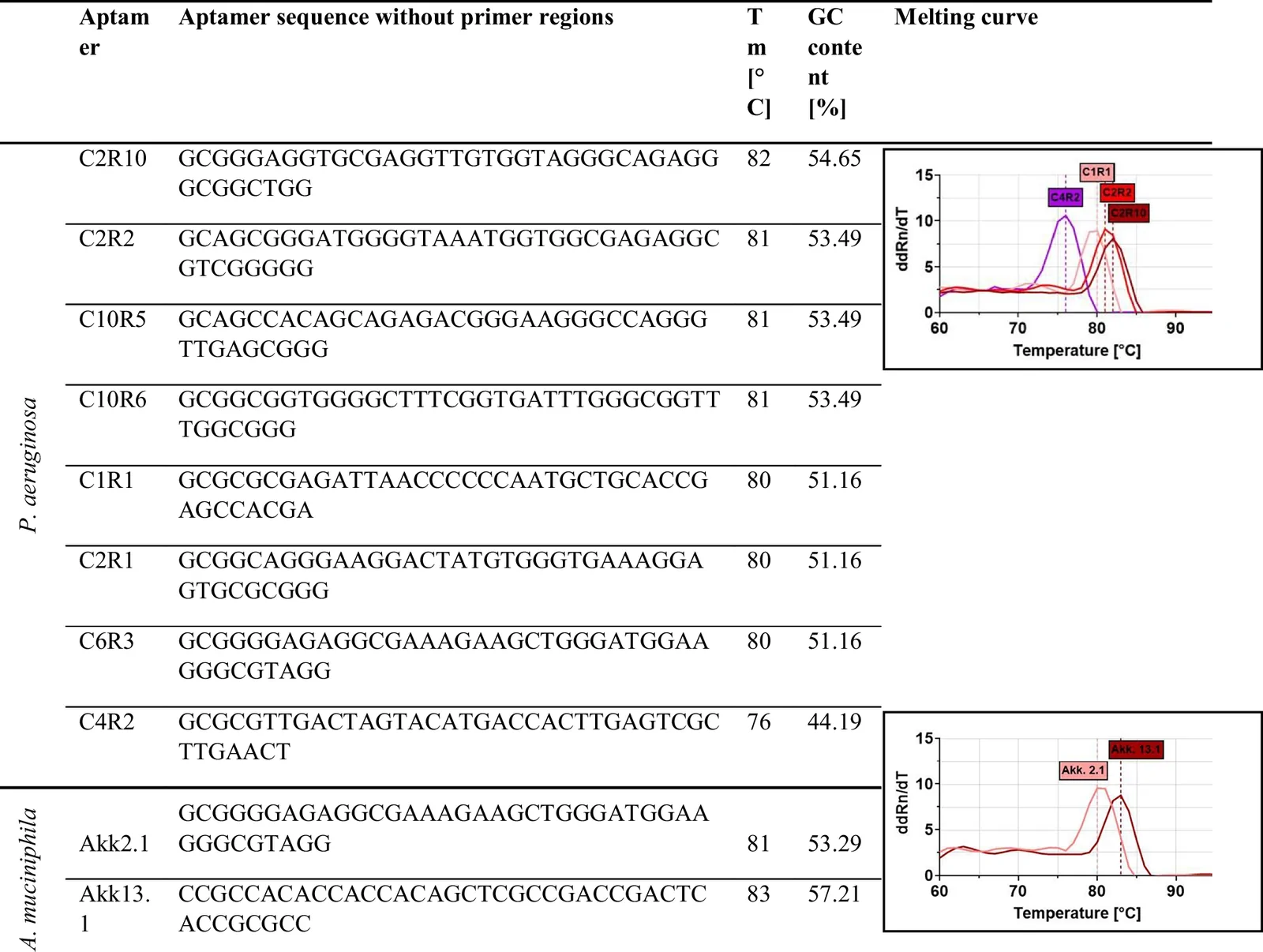
Overview of the single aptamers of *P. aeruginosa* (C2R10, C2R2, C10R5, C10R6, C1R1, C2R1, C6R3, C4R2) and *A. muciniphila* (Akk2.1, Akk13.1)

Melting curves of the respective single aptamers analyzed by quantitative PCR using the qTOWER^3^G touch (Analytik Jena, Jena, Germany) are shown on the right. ddRn/dT describes the change in fluorescence divided by the change in temperature. From the eight individual *P. aeruginosa* aptamers, four individual aptamers were shown as examples in the figure (right side)

## Discussion

As summarized in Table 2, the IMPATIENT qPCR results of all our SELEX projects include the details of the analyses on the respective *A. thaliana* root and *Candida *spec. SELEX processes, which inspired us to apply the qPCR including the melting curve analysis to all our previous SELEX libraries and to suggest IMPATIENT-qPCR as a general tool to estimate the success of aptamer evolution. IMPATIENT-qPCR has developed into a standard technique in our laboratory in all current projects and has proven its potential to estimate also promising end points of a SELEX and to judge effects of measures to increase selection pressure by counterselection, harshness of binding or washing conditions within the experiments. To our knowledge, it is the only PCR-based quality measurement for polyclonal libraries representing distinct SELEX rounds, which can be directly applied to eluted aptamers, without expenditure of additional precious target material for binding assays used in the FluCell- or FluMag-SELEX, respectively.

**Table 2:** Overview of published functional aptamer libraries originating from successful SELEX and analyzed by IMPATIENT-qPCR

Aptamer	SELEX rounds	*T*_m_ (°C) (start)	*T*_m_ (°C) (end)	Published
*P. aeruginosa*	16	64	80	Kubiczek et al. (2020)
*A. muciniphila*	13	64	81	Raber et al. (2021)
*P. distasonis*	14	63	82	Xing et al. (2021)
Apo- and holo-RBP4				
Apo	8	64	80	Kissmann et al. (2022a)
Holo	8	64	80	
*R. intestinalis*	7	63	80	Xing et al. (2022a, b)
*B. producta*	14	63	81	Xing et al. (2022a, b)
*Candida* spec	8	63	83	Kneißle et al. (2022)
*A. thaliana* roots				
Root tips	7	63	80	Kissmann et al. (2022b)
Root centers	7	63	80	
*R. microfusus*	13	63	74	Zhang et al. (2023)

IMPATIENT-qPCR is fast and allows the direct estimation of SELEX success and thus can represent a considerable acceleration of SELEX processes per se, which may even improve the potential of this unique technology to isolate high-affinity aptamers for a range of applications including diagnostics and biosensing. To constantly optimize the speed of aptamer evolution may inspire new scientists to take aptamers under severe consideration as potent and available binding molecules with a broad application potential. Speed is also of importance for the development of ligands in diagnostic applications immediately upon occurrence of health-threatening agents like the SARS-CoV-2 virus in the last pandemic, in which aptamers have already played a role (Aspermair et al. 2021; Reiner-Rozman et al. 2021; Kissmann et al. 2022a; Wang et al. 2022; Lou et al. 2022).

## Data Availability

Data will be made available on reasonable request.
